# The Evaluation of Hepatitis C Virus Core Antigen in Immunized Balb/C Mice

**DOI:** 10.5812/hepatmon.6141

**Published:** 2012-06-30

**Authors:** Elham Torbati, Mojgan Bandehpour, Parviz Pakzad, Nariman Mosaffa, Ameneh Koochaki, Bahram Kazemi

**Affiliations:** 1 Microbiology Department, Islamic Azad University, North Tehran Branch, Tehran, IR Iran; 2 Cellular and Molecular Research Center, Faculty of Medicine, Shahid Beheshti University of Medical Sciences, Tehran, IR Iran; 3 Biotechnology Department, Faculty of Medicine, Shahid Beheshti University of Medical Sciences, Tehran, IR Iran; 4 Immunology Department, Faculty of Medicine, Shahid Beheshti University of Medical Sciences, Tehran, IR Iran

**Keywords:** Hepacivirus, Core protein, Recombinant Proteins

## Abstract

**Background:**

Hepatitis infection represents one of the important causes of morbidity and mortality in developing countries, however there is not any effective vaccine against hepatitis C which is one of the significant problems in vaccine project.

**Objectives:**

The aim of the present study is to evaluate the role of HCV core protein in inducing IFN-Gamma secretion and TCL activities as a vaccine in Balb/C mice.

**Material and Methods:**

Our previous cloned plasmid (HCV Core gene into pETDuet-1) applied for protein expression in bacteria. The expressed and purified recombinant protein together with Freund’s adjuvant was injected to 15 Balb/c mice. The total IgG and IgG2a of immunized mice sera were evaluated after a week. Two weeks after booster injection, we studied the proliferation and IFNγ secretion of spleens, inguinal and popliteal lymph nodes lymphocytes by ELISA and ELISPOT.

**Results:**

The FSFC (Frequency of spot forming cells) of secreting cells of immunized mice with HCV/Core protein and sera IgG2a were considerably higher than the control groups.

**Conclusions:**

The core protein together with proper adjuvant can be a candidate vaccine against of HCV infection.

## 1. Background

Hepatitis infection represents one of the most important causes of morbidity and mortality in developing countries and is recognized as the leading cause of liver disease globally [1][2][3]. It is the main causative agent of persistent post transfusion hepatitis and has an increased risk for development of liver cirrhosis and hepatocellular carcinoma (HCC) [2][3][4][5]. The universal prevalence of HCV sero-positive patients is approximately 3% (170million individuals), and an estimate of 53000 deaths per year caused due to HCV globally [6][7][8][9]. The Hepatitis C Virus is capable to escape from host innate and adaptive immune system in immune competent patients, and in majority of people it is able to set up chronic infection which often persists despite the generation of virus-specific antibodies and T-cell responses [2][10].

The treatment of hepatitis C is not successful in most cases; furthermore, it is a very expensive and time consuming procedure, to this purpose, we as well as other researchers are interested to develop an HCV vaccine to prevent the infection [11][12]. HCV core protein was considered as immunogenic counterpart of the vaccine. Serum antibodies against HCV core protein epitopes (residues 7-21, 31-45, 49-63, 99-113) have been demonstrated in HCV patients. Due to contribution of all amino acid residues of the molecule to make perfect conformational epitopes, total of 191 amino acids of the molecule were included in the vaccine preparation in this study. The mentioned characteristics of the core protein and the conservative feature of the core gene in different HCV genotypes and the low susceptible of mutation in this region, make this protein an ideal candidate for HCV vaccine [12][13][14][15][16][17][18]

Hence, HCV-core specific T cells secreting IFNg can play a major role in prevention and treatment of the HCV-infection [15][19][20][21].

## 2. Objectives

Since the Core protein is a highly conserved protein, in this study, the efficiency of cellular immune responses in mice inoculated with core protein of Iranian HCV isolate were studied in order to lay a foundation for HCV core protein vaccine development in the future for regional population.

## 3. Materials and Methods

### 3.1. Plasmid Construction

PETDuet-1 expression vector containing HCV core fragment (573bp) were constructed in our previous study, were used in this research (in press). We used patient serums to generate the HCc PCR product by using below primers:

CoreF 5’CATATGAGCACACTTCCAAAACCCC3’,

CoreR 5’CTCGAGCGCGGAGGCTGGTGTTGTAAG3’,

the Core fragment was sub cloned into NdeI/XhoI cloning site of pETDuet-1 expression vector, in order to express HCV core gene [4].

### 3.2. Confirmation of Plasmid Construction

The Integrity of construct was confirmed by restriction enzyme analysis. The plasmid construction (pET-Duet1 containing the core fragment) was confirmed by specific Restriction enzymes which have a specific restriction site inside the inserted fragment, without any restriction site on the plasmid (SIGMA,USA).

The existence of the fragment in the pETDuet-1/core construct was confirmed by using polymerase chain reaction (PCR) and Core specific primers. PCR carried out in total reaction volume of 20 μL containing 0.1 μg of plasmid, 0.1 mM dNTPs (Fermentas, Lithuania), 1.5 mM MgCl2, 20 pmol of each forward and reverse primers and 1.25 units of Taq DNA polymerase (Fermentas, Lithuania), using the following PCR program: initial denaturation at 95°C for 3 minutes and 30 amplification cycles consist of denaturation at 94 ˚C for 30 second, annealing at 58 ˚C for 60 second and elongation at 72 ˚C for 90 second. Final extension was 72 ˚C for 5min.

A 573 bp PCR product was analyzed by electrophoresis on 1.5% agarose gel, stained by SYBR green II and visualized under a UV trans illuminator.

### 3.3. Protein Expression

Recombinant plasmid was transformed into Nova Blue Competent Cells (Novagen) and plated onto Luria-Bertani (LB) (Merck, Germany) agar plates containing 100 µg/mL of Ampicilin (SIGMA, Germany). The protein expression was carried out as mentioned [21][22][23].

### 3.4. Protein Purification

The supernatant equilibrated in a solution containing 2M Urea (Merck, Germany), 50 mM Tris-HCl (Merck, Germany), pH:8, and 2mM EDTA (Merck, Germany), and loaded on to a S-Bind resin affinity chromatography column (Novagen,) , the flow-through collected, then the column washed with 20 mL of the same buffer and recombinant protein was eluted into 2 mL of Elution Buffer containing 2M Sodium Thiocyanate (Fermentas, Lithuania) and 2mM EDTA (Merck, Germany). Fractions of 1.5 mL were collected. The purified recombinant proteins were dialyzed against Sucrose. Finally the protein concentration was determined by spectrophotometer.

### 3.5. SDS-PAGE and Western Blotting

Cell lysate and purified protein were electrophoresed through 12% SDS-PAGE gel and electrophoretically transferred in to a nitrocellulose membrane [22]. The bands fixed and soaked for 30 minutes in a 10% gelatin solution on shaker (80 rpm) and bound to anti-S-tag alkaline phosphates conjugated antibodies (1:5000) (Novagen) the bands were visualized by adding the Alkaline Phosphates substrate (NBT/BCIP) (Novagen).

### 3.6. Immunization of BALB/c Mice

Since there is a great similarity between the BALB/c mice and human immunity system mechanisms, identical (gender, age, weight, etc.) mice were used in order to minimize the rate of false results.

Female BALB/c mice were housed in approved animal care facilities during the experimental period . According to the Guiding Principles in the Care and Use of Laboratory Animals, the immunization performed as follow:

### 3.7. Negative Controls

A group of 6 female 6-8 weekold BALB/c mice, 18-20 g of weight (Razi. Institute of Iran) were immunized with a total of 150 μL of Phosphate-Buffered Salin-1X (PBS) , split in to 3 footpad injections done with 2 weeks intervals as a mocked-immunized group.

### 3.8. Test Group

A group of 10 female 6-8 week old BALB/c mice, 18-20 g of weight (Razi. Institute of Iran) were injected in foot pad with 50 ng of recombinant protein in complete Freund’s adjuvant (SIGMA, Germany) on the first day and boosted 2 weeks later with the same amount of protein in incomplete Freund’s adjuvant (SIGMA, Germany) and on day 28, the mice were immunized with 50ng of the protein in PBS. On day 42- before isolation of splenocytes and popliteal and inguinal lymph nodes-serum samples were obtained by retro-orbital puncture.

Freund’s adjuvant is a general adjuvant used for evaluation of peptide antigenicity in animal studies which could be substituted with proper adjuvant in human studies.

### 3.9. Determination of Antibody Production in Serum and Lymphocytes of Spleens and Lymph Nodes

Blood samples of immunized mice were collected 7 and 14 days after immunization. Pre-immune sera were considered as controls. The total IgG, IgG2a antibodies against the HCc protein was detected using mouse IgG2a and IgG ELISA quantization kit (Bethyl, TX).

### 3.10. Ex Vivo Direct Enzyme-Linked Immunospot (ELISPOT) Assay for IFN-γ

ELISPOT assay for detecting IFN-γ secreting cells specific for HCV core epitopes has been formerly described by Duenas-Carrera et al. [24] , 96 PVDF-bottomed well plate (multiscreen filter plate, Millipore, Cat No. MAIPS45510) were coated with 100 μL murine IFN-γ-specific antibody (Mouse IF gamma ELISPOT Ready-SET-Go, bioscience, Cat No.88-7384) and the test performed according to the kit instructions. The plates were dried and kept in the dark; finally spots were counted under dissection microscope (Zisse, Germany).

### 3.11. The MTT Assay

Cells (5×103 cells/well) were plated onto 96-well plates and cultured for 24, 48 and 72 h, then 100μL of MTT (Sigma,USA) (5 mg/mL MTT ,90 μL RPMI) added to each well and shook at 37 ˚C for 4 hours, then centrifuged at 1000 g for 5 minutes . The supernatant decanted and 200 μL/well of Dimethyl sulfoxide (DMSO) (SIGMA, Germany) was added. The stained cells were eluted with methanol (Merck, Germany) and the optical density of each well was measured at 590 nm using the microplate reader (Sunrise Tecan, Austria) [24].

### 3.12. Dot Blot

Dot blot is an approval and quality test which evaluates specific anti bodies production in response to HCV-Core protein immunization. Recombinant proteins blotted on nitrocellulose membrane (Porablot, Germany), then the blots were subjected to primary (Immunized sera) and secondary antibody (human anti-IgG peroxidase conjugated), subsequently detected by adding substrate (30% H_2_O_2_ and DAB (Merck, Germany)) diluted in 1 M Tris (Merck, Germany) and TBS) [20].

### 3.13. Statistical Analysis

All statistics of the study were recorded as computerized database and a descriptive statistical analysis (ANOVA) was performed using the SPSS Software (SPSS, version 12.0).Differences were considered statistically significant (P < 0.05).

## 4. Results

### 4.1. Confirmation of Constructed Plasmid

**Figure 1:**
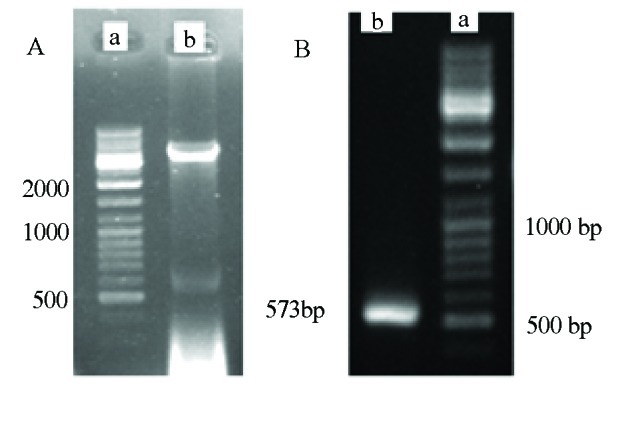
A) Clone Confirmation, a) 100bp DNA ladder, b) 573bp Digested Insert by NdeI and XhoI; B) 573bp Core PCR Product on 1.5 % Agarose Gel

Integrity of construct was confirmed by restriction analysis with NdeI and XhoI enzymes. The existence of an inserted fragment was confirmed by this method (Figure 1). The PCR product was also subjected to electrophoresis in 1.5% agarose gel, stained by SYBR green II and visualized under a UV Trans illuminator and photographed. The 573 bp band was observed which confirmed core gene amplification (Figure 1).

### 4.2. Gene Expression and Protein Purification

**Figure 2:**
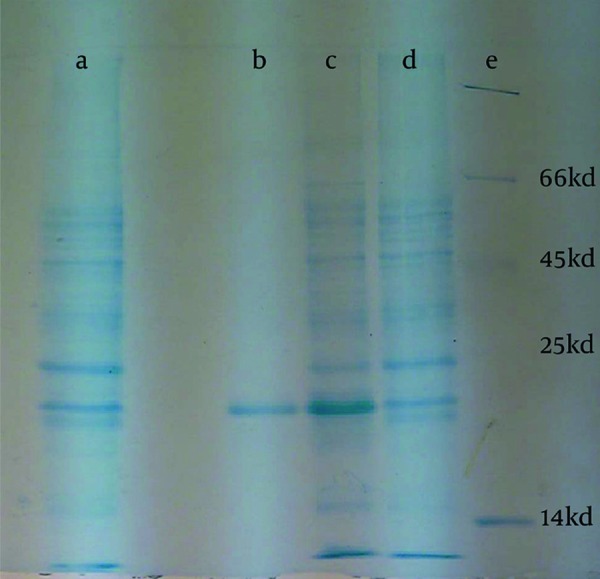
12%SDS-PAGE gel, (a) Negative Control, (b) Purified Protein (c) the Bacterial Lysate Expressing the Recombinant Core Protein (d) Nova Blue Cells Without the Recombinant Plasmid, (e) Protein Ladder

**Figure 3:**
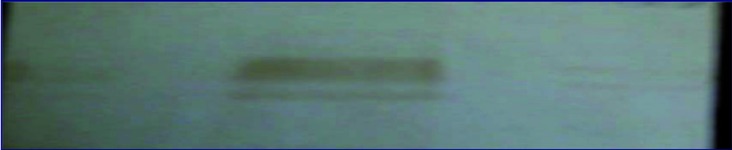
Western Blot Analysis of Core Protein

The Core gene expression confirmed by electrophoresis on 12% SDS-polyacrylamide gel and Coomassie Brilliant blue R250 staining, a 21 Kd band observed while no band observed as negative control (cells without recombinant plasmid) (Figure 2). The O.D of the collected protein fractions were read by using spectroscopy apparatus at 280 nm and recombinant protein purification confirmed, the eluting solution were subjected to evaluation by electrophoresing through SDS-PAGE gel followed by Coomassie brilliant blue R250 (SIGMA,USA) staining finally a 21 KD band observed (Figure 2), then HCV Core protein was detected using Western blotting technique (Figure 3).

### 4.3. Antibody Detection in Immunized Mice by ELISA

**Figure 4:**
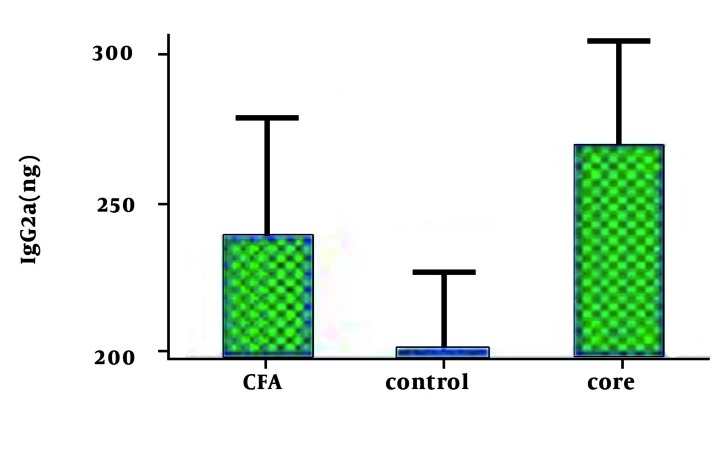
IgG2a Production in Immunized Mice in Comparison With Control Groups

The total IgG and IgG2a levels against HCc protein were significant compared to the control groups (P < 0.01) (Figure 4).

### 4.4. Ex vivo Direct Enzyme-Linked Immunospot (ELISPOT) Assay for IFN-γ

**Figure 5:**
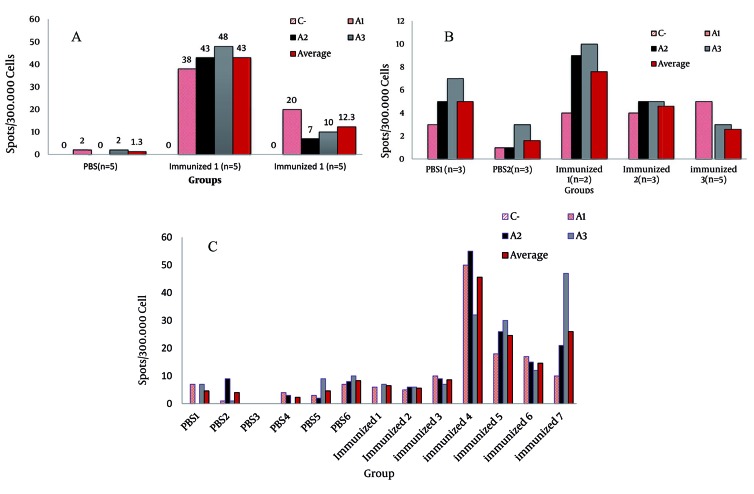
Induction of Cellular Immune Response by Injection of HCV Core Protein to BALB/C Mice A) The Quantity of HCV Core Specific T Cells Expressing IFNg in Popliteal Lymph nodes in Comparison to the Negative and Positive Controls, B) The quantity of HCV Core Specific T Cells Expressing IFNg in Inguinal Lymph nodes in Comparison to the Negative and Positive Controls, C) The Quantity of HCV Core Specific T Cells Expressing IFNg in Splenocytes in Comparison to the Negative and Positive Controls

Th1 cytokine IFN-γ secreting cells were detected, 2 days after stimulation with 1 µg/mL recombinant HCV/Core protein. The frequency of spot forming cells (FSFC) indicated the spot number per 300,000 well. The HCV/Core immunized mice illustrated higher FSFC than the negative controls (Figure 5). FSFC of popliteal was significantly higher than the FSFC of inguinal and splenocytes which may strongly is related to the site of injection.

Overall, immunized mice showed higher sum of spots than the control group. The popliteal lymph nodes of immunized mice illustrated larger quantity of HCV Core specific T cells that secreted IFNg.

### 4.5. MTT Assay

The reproducibility of the MTT assay was tremendously good .triplicate wells generally gave values within 2% of the mean and the yellow color of tetrazolium converted to the violet (formazan) which is the sign of the viability and proliferation of the cells, the O.D at 590 nm was measured, the O.D values of test group were significant higher than those of negative controls (P < 0.01).

### 4.6. Dot Blotting

The dots were visualized by adding DAB and 30% H_2_O_2_. the sera of PBS group (Negative control) has not formed any dot, the sera of test group illustrated dot formation at two different concentrations (1:100 and 1:500) which approved the response of hommoral immune system of immunized mice against HCV core protein.

## 5. Discussion

Knowledge of the HCV proteins roles has notably heightened our awareness of the possible HCV-mediated biological functions in infected hosts. HCV causes silent infection and the data suggested that continued existence of HCV in infected cells would probably be destructive for host cells moreover, the core protein has been detected in chronically infected HCV patients [6] .In its approval, Alekseeva et al. suggested that the administration of highly expressed HCV core protein, as well as frequent core protein injections possibly will hinder core-specific immune response [22]. Duenas-Carrera et al. explained that HCV core antigen (HCcAg) is an important object to develop HCV remedial and preventive approaches [2]. HCV core protein is associated with a wide range of cellular proteins and manipulates various host cell functions to cause disease [22]. The HCV core protein is a multifunctional protein involves in many processes; it is phosphorylated and has both cytoplasmic and nuclear localization, and as a result it may possibly have several roles in the viral life cycle. Moreover, previous studies suggested HCV core protein regulatory functions in viral and cellular proteins and declared this protein have transformation activities as well [6][18][24].One of the difficulties facing in development of HCV vaccine is their broad genetic variant, according to the features of HCV core protein, the HCcAg is a highly conserved protein [2][6][8] .Hittomi et al. reported that the analysis of Core gene sequences isolated from samples obtained from HCV-infected patients confirmed considerable conservation of C gene sequences. Such conservation of C gene sequences is essential to achieve an HCV vaccine against the Core gene product [25].

It has also been reported that in primary HCV infection, a humoral immune response, naturally results to the production of isolate-specific neutralizing antibodies. These antibodies merely attach to native and not denatured proteins, which suggested that they identify conformational epitopes. The fact that a large number of patients with anti-HCV antibodies harbor a replicating virus, are infectious, and evidence of liver injury ,implies that antibodies are not protective, as a result, they are not proper candidates for HCV vaccine evaluations [25].

During chronic infection with HCV, a loss of CD8+and CD4+ reactivity in the blood against a range of peptides is well documented, in contrast to patients who infection has been resolved infected individuals with persistent viremia and chronic liver disease have less PBMC showing type 1 cytokine (IL-2, IFN-γ) responses to HCV core protein than self-limited HCV patients [6]. Additionally, in HCV-infected patients, no mutation was found in Core gene despite the presence of T cells expressing IFNg specific for these epitope, suggesting that immune responses to HCV core antigen did not lead to the appearance of escaped variants [2]. Koziel et al. reported that HCV-specific T-cells cytolytic activity is present in bulk-expanded liver-infiltrating lymphocytes in the absence of any in vitro antigenic stimulus, whereas such activity could not be detected in bulk-expanded PBMC, which suggest a tissue-specific localization of HCV-specific CTL [15]. Moreover, Paliard et al. (2010) suggested that HCV-specific T cells expressing IFNg persevere for a long time following immunization and in patients who have resolved acute infection [8]. The examined pattern of a dynamic CTL response and feebler humoral responses causing disease, declared that HCV persistence relates to the pattern of cytokine release [25]. There is a rising amount of proofs demonstrating that a vaccine eliciting both Core-specific CD41 and CD81 T cells may well have a therapeutic value [8]. It has also been reported that the HCV core protein induces sensitization of both CD4+and CD8+ lymphocytes [2]. Although HCV-specific CTL may mediate liver damage in chronic infections, it remains possible that HCV-specific CTL contributes to either clearance of viremia or lack of progression to chronic disease in some individuals. Since HCV-specific CTL is shown to be important in limiting viral replications, Immunization with peptides alone has been shown to lead to the induction of a protective CD8+ CTL response [19][24]. IFNs are powerful antiviral cytokines, among these Cytokines, IFN-γ implicates in CD4+ and CD8+ T-cells responses as well as NKs which are fundamental for virus clearance in infected patients. Hence, in this study we assessed the amount of IFN-γ secreted by immunized mice T-Cells, which is directly related to the active T cells expressing IFNg in immunized mice. The work described in this paper tries to find out and evaluate the core protein and its function as object to induce HCV-specific CTL responses, for this we expressed the HCV core gene from Tehran HCV isolates, employing pETDuet-1 expression vector and Nova Blue Prokaryotic cells and the purified protein directly assessed and quantified by standard protein methods including SDS-PAGE, Western Blot and Nano drop Spectrometry and etc. Accordingly, we evaluated the CTL activity of the BALB/C mice immunized with the recombinant HCV core protein. In this study, we measured ex vivo immune responses by IFN-γ enzyme-linked immunosorbent spot assay (ELISPOT) and proliferation assay by means of established robust technique and identified responses against core protein to assess the Balb/C mice cellular immunity responses.

We observed that the popliteal lymph nodes of the immunized mice showed increased amount of T cells expressing IFNg and IFN-γ secretions which are closely related to the injection site (footpad). With reference to the results of present study and by means of mentioned techniques which are highly sensitive and specific, we observed that CTL responses and IFN-γ secretion is increased in the immunized mice with recombinant HCV core protein.
